# Acute Hemoperitoneum Caused by the Ruptured Arc of Buhler

**DOI:** 10.7759/cureus.79145

**Published:** 2025-02-17

**Authors:** Devin Smith, Lucas N Canaan, Bradley Kuhn

**Affiliations:** 1 Department of Surgery, Northeast Georgia Medical Center Gainesville, Gainesville, USA; 2 Department of Trauma and Acute Care Surgery, Northeast Georgia Medical Center Gainesville, Gainesville, USA

**Keywords:** abdominal anatomy, arc of buhler, emergency abdominal surgery, general and vascular surgery, resusitation

## Abstract

The arc of Buhler is a persistent connection between the celiac trunk and the superior mesenteric artery, which is an embryogenic remnant. This case report details the series of events that led to the discovery of an arc of Buhler after a patient was found to have significant hemoperitoneum secondary to the rupture of this pathology. It includes the patient's initial presentation, resuscitation, and surgical management.

## Introduction

The arc of Buhler is a rare anatomical variant characterized by an anastomosis between the superior mesenteric artery (SMA) and the celiac artery. While not commonly observed in the general population, this vascular connection plays a role in the collateral circulation of the abdominal viscera, particularly in scenarios involving arterial occlusion or impaired blood flow. The prevalence of the arc of Buhler is variable in the literature, ranging from 1% to 5% [1,2]. Its presence carries considerable significance, particularly in the context of abdominal surgeries, for preoperative planning in procedures such as pancreaticoduodenectomy and in assessing patients presenting with nontraumatic, large-volume hemoperitoneum [2].

Beyond its relevance for preoperative planning in both laparoscopic and open procedures, the arc of Buhler is associated with certain risks. One such risk, as evidenced in our patient, is the development of an aneurysm or pseudoaneurysm involving the arc. The primary concern with this pathology is rupture, which can result in life-threatening, uncontrolled intra-abdominal hemorrhage. Although complications related to the arc of Buhler are rare, a 2019 case report documented a fatal rupture of a pseudoaneurysm. The patient, who presented with massive retroperitoneal hemorrhage following rupture, suffered severe blood loss despite resuscitative efforts. Although the patient did reach the operating room, the condition deteriorated, and subsequent disseminated intravascular coagulation led to cardiac arrest the following morning [3]. This case report will examine a spontaneously ruptured arc of Buhler and provide a detailed discussion of patient care, including initial evaluation, resuscitation, and surgical management.

## Case presentation

A 60-year-old patient initially presented through the emergency department from an outside facility via helicopter. The initial report provided to the surgical team indicated that the patient had experienced a syncopal episode at home, which was preceded by burning pain in the epigastric region, nausea, vomiting, diaphoresis, pallor, and hypotension, as reported by emergency medical services. At the outside facility's emergency department, the patient was found to be progressively hemodynamically unstable, leading to intubation and the initiation of vasopressor support.

**Figure 1:**
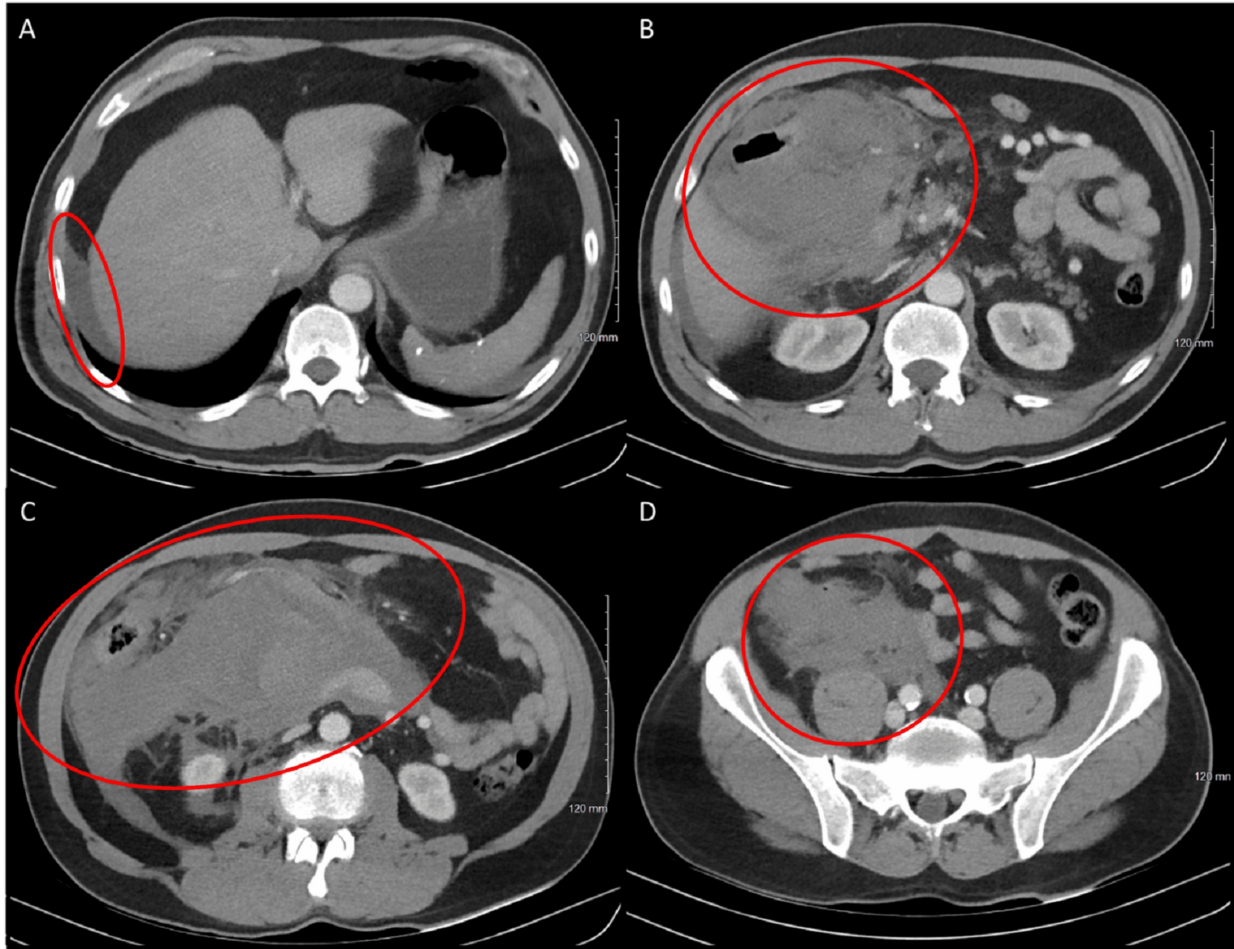
Axial cuts through a CT abdomen pelvis with intravenous contrast, demonstrating free fluid within the abdomen. (A) Free fluid along the right lateral edge of the liver (red circle). (B) Free fluid in the mid-upper abdomen at the inferior edge of the liver (red circle). (C) Free fluid in the lower abdomen (red circle). (D) Free fluid within the right hemipelvis (red circle) CT: computed tomography

Initial management at the outside facility included resuscitation with blood products, including two units of fresh frozen plasma, two units of packed red blood cells, one unit of pooled platelets, and four units of whole blood. Upon arrival at a community level 1 trauma center, the surgical team evaluated the patient. This evaluation included reviewing a computed tomography (CT) scan completed at the outside hospital. There was a concern for spontaneous bleeding from a major branch of the celiac or SMA versus heavy bleeding from a large vein. A multilumen access catheter was placed in the patient’s right internal jugular for high-volume blood product resuscitation in the operating room, and the patient was prepped for an emergent exploratory laparotomy. A focused assessment with sonography in trauma exam was performed in the emergency department and was positive, consistent with the findings on the CT imaging from the outside facility. CT imaging is demonstrated in Figure 1 with the presence of a large volume of hemoperitoneum focused in the right hemiabdomen.

Following initial treatment in the emergency department by the acute care surgery team, the patient was urgently taken to the operating room for evaluation. His abdomen was accessed through a laparotomy incision. Upon entering the abdomen, a large mesenteric hematoma was encountered. This hematoma was causing local abdominal compartment syndrome by compressing the inferior vena cava. Further evaluation of the mesentery revealed a vascular anomaly within the mesentery of the right colon. A right hemicolectomy was performed, and the patient was left in discontinuity. Due to the patient's continued hemodynamic instability and ongoing vasopressor support, it was decided to proceed with damage control surgery. After appropriate control of the artery, no signs of persistent bleeding were noted. The abdomen was packed, and an ABThera (3M, Saint Paul, MN) was placed. The patient was then taken to the surgical and trauma intensive care unit (STICU) and intubated for continued resuscitation.

In the STICU, the patient was placed on a 3% normal saline drip for 24 hours to assist with bowel edema. The morning following his initial surgery, the patient was extubated without issue. At the time of extubation, he continued to be on vasopressor support, specifically vasopressin. Three days after his initial surgery, he was planned to return to the operating room for anastomosis and definitive closure.

**Figure 2:**
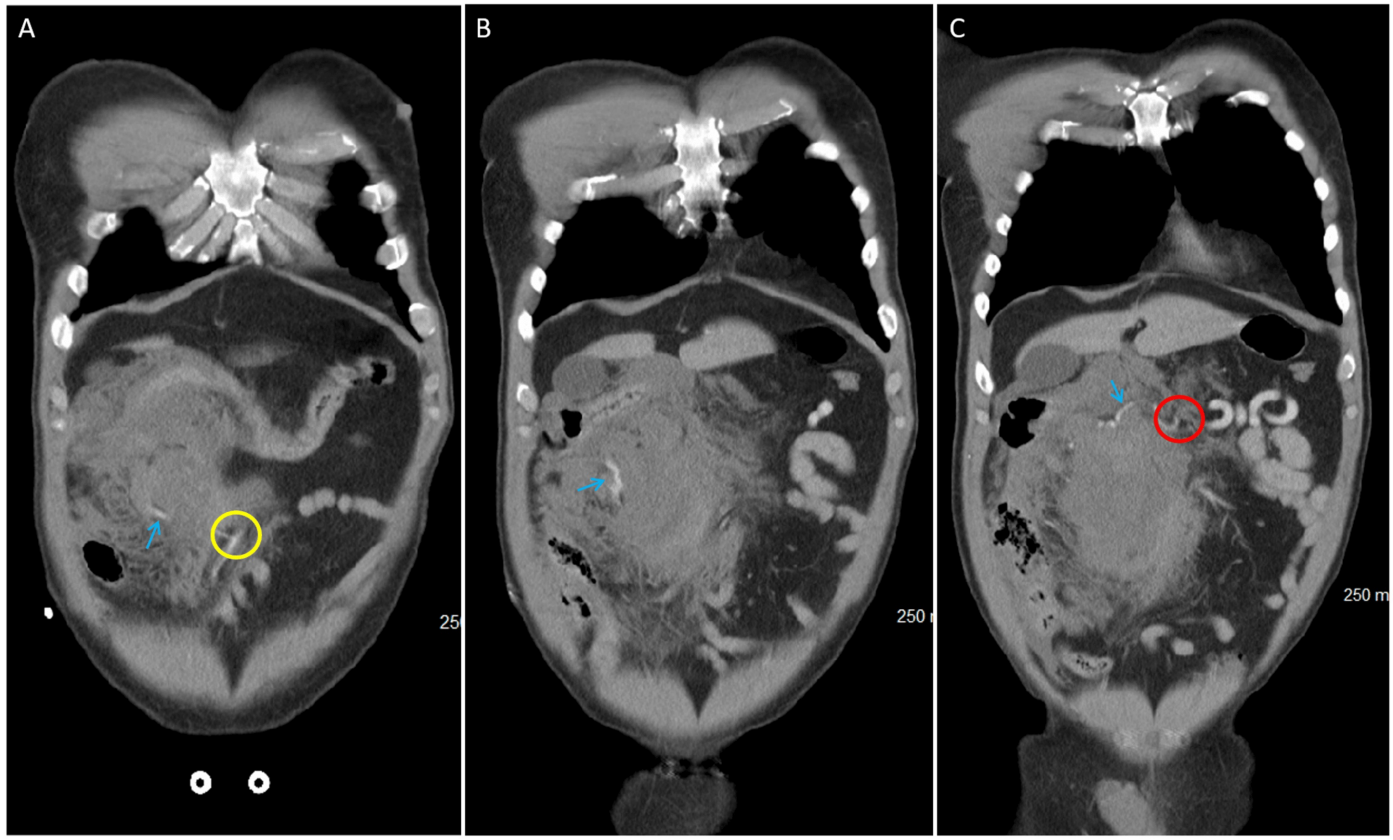
Coronal CT images from anterior to posterior, demonstrating the course of the arc of Buehler through the abdomen. (A) The anteriormost slice of the coronal imaging, showing the distal anastomosis to the SMA represented by the yellow circle with the arc of Buhler indicated by the blue arrow. (B) The mid-abdominal coronal imaging, demonstrating the course of the arc of Buhler as it progresses proximally indicated by the blue arrow. (C) The posteriormost slice from coronal CT imaging, demonstrating the proximal anastomosis with the celiac trunk indicated by the red circle with the path of the arc of Buhler indicated by the blue arrow Yellow circle: the arc of Buehler connection to SMA Red circle: the arc of Buehler connection to the celiac trunk Blue arrow: the arc of Buehler as it progresses through images CT: computed tomography; SMA: superior mesenteric artery

During the take-back procedure, no signs of additional bleeding were observed. Close inspection of the artery associated with the initial bleed was highly suspicious for a Buehler variant with an aneurysmal rupture. In the review of original imaging, although difficult to trace in its entirety, the arc of Buehler can be traced from its origin at the celiac trunk to the SMA. This is best detailed in Figure 2. An ileocolonic anastomosis was created between the terminal ileum and the proximal margin of the colon. Drains were placed, and the abdomen was closed. The patient ultimately spent two more days in the surgical and trauma ICU, after which he was transferred to the floor. He had no further issues on the surgical floor and was ultimately discharged without complication. His total time in the hospital was eight days.

## Discussion

The arc of Buhler, a rare anatomical variant, is a collateral vessel that connects the celiac trunk and the SMA, creating a bridge between two major vessels of visceral circulation. Its prevalence is estimated at approximately 1%-5% of individuals, while other sources comment it is closer to 1%-4% [1,2]. Considering the vessels' connection to large arterial structures, hemorrhage would pose a life-threatening risk in the event of rupture. In this case, the rupture of the arc of Buhler led to significant intra-abdominal bleeding and hemodynamic instability.

Generally, the arc of Buhler is described as a collateral vessel between the celiac artery and SMA and is often present in the instance of celiac trunk stenosis [2,3]. In the absence of the arc of Buhler, the gastroduodenal artery, pancreaticoduodenal arteries, and dorsal pancreatic artery are the key anastomoses between the celiac artery and mesenteric artery [2]. Hemoperitoneum due to vascular rupture is an uncommon cause of abdominal hemorrhage, with typical etiologies including trauma, ruptured aneurysms, or ruptured ectopic pregnancy [1]. Given the low prevalence of the arc of Buhler, hemoperitoneum secondary to this vessel is even rarer. Clinical manifestations often resemble those of other intra-abdominal emergencies caused by bleeding, requiring a high index of suspicion and advanced imaging techniques for accurate diagnosis.

In this case, contrast-enhanced CT angiography was critical in identifying the ruptured arc of Buhler. Due to its rapid speed and availability, CT imaging is often the first-line modality for evaluating concerns related to bleeding from visceral vessels [4]. Although the initial imaging suggested the presence of an arc of Buhler, this variant was not definitively confirmed until the abdomen was explored.

The management of a ruptured arc of Buhler typically depends on the hemodynamic stability of the patient. In cases where hemorrhage is controlled and the patient remains stable, endovascular embolization is a viable option [5]. Embolization techniques are often better tolerated by patients as they are far less invasive than open procedures. That being said, embolizing will cease to flow through the anastomosis created by the arc of Buhler [5]. In the setting of a large hemoperitoneum with hemodynamic instability, this made the decision more clear, and the surgical team proceeded to open surgery.

The importance of recognizing and understanding variations in vascular anatomy cannot be overstated, particularly in managing unexplained hemoperitoneum. While the arc of Buhler is rare, with estimates of around 1.7% of the population, its rupture should be considered when standard causes of bleeding have been ruled out, especially in patients without a clear traumatic history [6]. Patients presenting in this manner should be managed similarly to those with intra-abdominal hemorrhage secondary to trauma: resuscitate with blood products, ideally in a balanced manner, followed by identification of the bleeding vessel, and then surgical management, either through embolization with vascular surgery or interventional radiology, or through open surgical management.

## Conclusions

This case report highlights the potentially devastating outcomes resulting from a ruptured arc of Buhler. Given its connection between two major vascular structures, rapid and severe hemorrhage can occur. While this is a relatively rare anatomical variation, it must be considered during surgical procedures, particularly in cases involving acute abdominal hemorrhage. Resuscitation should follow standard protocols, focusing on balanced resuscitation with blood products and correction of coagulopathies, followed by surgical intervention for ligation of the bleeding vessel. Beyond acute rupture scenarios leading to hemoperitoneum, it is essential for surgeons to be mindful of this anatomical variant in routine practice to avoid inadvertent damage to the vessel, which can result in significant bleeding.
